# Prevalence of Mental Disorders among Prisoners in the State of Sao Paulo, Brazil

**DOI:** 10.1371/journal.pone.0088836

**Published:** 2014-02-14

**Authors:** Sergio Baxter Andreoli, Maíra Mendes dos Santos, Maria Ines Quintana, Wagner Silva Ribeiro, Sergio Luiz Blay, Jose Geraldo Vernet Taborda, Jair de Jesus Mari

**Affiliations:** 1 Psychiatry Department, São Paulo Federal University, São Paulo, Brazil; 2 Santos Catholic University, Santos, Brazil; 3 Department of Internal Medicine, Porto Alegre Science of Health Federal University, Porto Alegre, Brazil; Emory University School Of Medicine, United States of America

## Abstract

**Objective:**

To determine the prevalence of psychiatric disorders in the prison population in the State of São Paulo, Brazil.

**Methods:**

Through stratified random sampling, 1.192 men and 617 women prisoners were evaluated for the presence of psychiatric disorders by the Composite International Diagnostic Interview, 2.1 version, according to definitions and criteria of International Classification of Diseases (ICD-10). The prevalence estimates of mental disorders and their respective 95% confidence intervals were calculated and adjusted for sample design through complex sample analysis.

**Results:**

Lifetime and 12-month prevalence rates differed between genders. Lifetime and 12-month prevalence of any mental disorder was, respectively, 68.9% and 39.2% among women, and 56.1% and 22.1% among men. Lifetime and 12-month prevalence of anxious-phobic disorders was, respectively, 50% and 27.7% among women and 35.3% and 13.6% among men, of affective disorders was 40% and 21% among women and 20.8% and 9.9% among men, and of drug-related disorders was 25.2% and 1.6% among women and 26.5% and 1.3% among men. For severe mental disorders (psychotic, bipolar disorders, and severe depression), the lifetime and 12-month prevalence rates were, respectively, 25.8% and 14.7% among women, and 12.3% and 6.3% among men.

**Conclusions:**

This is the first large-scale epidemiological study performed with the prison population in Brazil, revealed high rates of psychiatric disorders among men and women. Many similarities, as well as some differences, were found between our results and those of studies conducted in other countries. The differences observed are more likely due to the peculiarities of the prison systems in each country than to the diagnostic criteria adopted in the studies. This fact reinforces the importance of conducting such studies as part of planning and development of appropriate policies for the particular mental health needs of specific prison populations.

## Introduction

Epidemiological studies conducted with prisoners in several countries have shown a high prevalence of psychiatric morbidity. The prevalence of severe mental disorders can be 5 to 10 times higher than in the general population [1]. In European prisons, the prevalence of psychotic disorders is around 5%, of depressive or anxiety disorders is around 25%, and of substance-related disorders is approximately 40% [2]. An extensive literature review done in 24 countries revealed rates of depression of around 10% and 14% in male and female prisoners, and approximately 4% of psychotic disorders in both genders [3].

Brazil has approximately half a million prisoners and the State of São Paulo has one of the largest prison populations in the country, approximately 40% of the national total. Nevertheless, information about prisoners’ health conditions is scarce. A few studies have reported high rates of health problems in Brazil’s prison system, including mental disorders. In a women’s prison in São Paulo, the prevalence of common mental disorders was reported as being 26.6% [4]. A study conducted in Salvador, in the northeast of Brazil, with 497 prisoners in closed and semi-opened systems reported high prevalence rates of mental disorders. The rates found for prisoners in closed systems were 5.2% for bipolar affective disorder, 17.6% for depression, and 1.4% for psychotic disorders. For prisoners in semi-open systems, the rates were 10.1% for bipolar affective disorder, 18.8% for depression, and 12.6% for psychotic disorders [5].

Knowing the mental health needs of prisoners is crucial in order for prison systems to develop appropriate health care programs for this population. Thus, establishing the prevalence rates of mental disorders is of great importance [6]. This study aimed to determine the prevalence of mental disorders in the prison population in the state of São Paulo, Brazil.

## Methods

### Ethics Statement

This study was approved by the ethical committee of the Federal University of Sao Paulo (CEP 1051/05) and the State of Sao Paulo Department of Penitentiary Administration (process n. CS 295/05). All participants signed an informed consent to participate in this study. Individuals that declined to participate were not disadvantaged in any other way by not participating in the study.

We conducted a cross-sectional study with a probabilistic sample of 105 prisons with closed regime (5 female prisons – FP, 4 female resocialization centers – FRC, 32 temporary detention centers for men – TDC, and 64 male prisons – MP).

Prisoners in custody who consented to participate in the study were included whereas those in maximum-security units, or in custodial hospitals, were excluded for reasons of operational difficulty. Ethnicity classification was determined by subject. It was delivered to the respondent a card containing the different ethnic groups in Brazil (used by IBGE - Brazilian Institute for Geography and Statistics). The survey was conducted from May 2006 to January 2007.

The sample was selected using multistage sampling with probability proportional to size. Five administrative regions of the State of São Paulo responsible for prison administration were considered as strata. Male inmates were randomly selected from four units drawn in each stratum (20 randomly selected units, 10 MP and 10 TDC). Female inmates were randomly selected from the nine prison units of the State of São Paulo (five prisons and four rehabilitation centers).

The following parameters were considered for calculation of the sample size: a) population size in each stratum, b) estimated prevalence of 2% and minimum acceptable frequency of 1%, c) confidence level of 95%, and d) estimated loss of 10%. Based on these parameters, a total of 2,320 interviews was foreseen. The final sample was formed of 1.192 men and 617 women (response rate 77.9%), with a loss of 26.8% for men and 10.5% for women. The main causes of sample losses were: difficult access to prisoners (n = 336), refusals (n = 135), prison transfer (n = 16), and errors in the identification data (n = 24). The interviewers or study coordinator had no direct access to the raffled interviewed, the invitation to participate in the study was conducted by a prison guard, and if the subject refuses to participate no other information was given about him/her to the team study.

### Instruments

The validated Brazilian version of the instrument “Composite International Diagnostic Interview” (CIDI), version 2.1, was used for diagnosis of psychiatric disorders [7] [8] [11]. CIDI is a standard structured questionnaire, designed to be used by trained non-clinician interviewers. It generates psychiatric diagnoses according to criteria of the International Classification of Diseases, 10th edition (ICD-10) and the Diagnostic and Statistic Manual of Mental Disorders, 4th edition (DSM-IV). Because ICD-10 is considered an efficient procedure for best-estimate diagnosis [9], in this study the lifetime and 12 month prevalence rates of mental disorders were generated by ICD-10. To estimate the prevalence of severe mental disorders, the following disorders were grouped: schizophrenic, delusional, acute psychotic, schizoaffective, severe depression and bipolar affective. The CIDI was applied by law interviewers trained and supervised by trainers from CIDI Brazil Official Center. The Portuguese version of the CIDI 2.1 has been previously validated and adapted to Brazil’s social and cultural context [7], [10], [11].

### Statistical Analysis

The prevalence estimates of mental disorders and their respective 95% confidence intervals were calculated and adjusted for sample design through complex sample analysis [12]. The analysis was different for each gender. For men, the administrative regions of the state and the type of prison (MP or TDC) were considered as strata. Since the number of woman recruited was to small by type of prison facility we decided to collapse the two groups in the analyses. Prison units drawn in the first stage and the inmates, who were randomly selected in the second stage, were considered as clusters. In the first stage, two units were drawn in each stratum and in the second stage, the number of prisoners drawn was proportional to the size of the stratum and without replacement. For women, the administrative regions and the prisons were considered as strata. The inmates were randomly selected in proportion to the size of the stratum and without replacement.

## Results

The characteristics of the prison population of the state of São Paulo are shown in Table 1. We can observe the predominance of white prisoners born in the state, with more than 5 years’ education, and who were employed before being arrested. Gender differences were observed: most women were single, had more children and were paid less than men. Differences regarding the prison regime were also observed. Most of the individuals in prisons were aged 28–47 years and were not working while incarcerated. Those in TDC were younger.

**Table 1:** Sociodemographic and criminal characteristics of the prison population in the state of São Paulo, Brazil (n = 1809).

Characteristics	Female population	Male population	
		Prisons	TDC^1^
	N (%)	N (%)	N (%)
**Worked before being arrested**			
No	240 (37.8)	166 (23.1)	94 (18.1)
Yes	377 (62.2)	510 (76.9)	422 (81.9)
**Marital status**			
Married	257 (39.9)	383 (56.2)	321 (64.9)
Single	356 (60.1)	292 (43.8)	188 (35.1)
**Number of children**			
0	100 (12.7)	207 (30.5)	204 (38.6)
1–2	274 (48.1)	293 (42.7)	221 (43.4)
3–4	174 (27.9)	122 (18.5)	73 (14.9)
5 or more	69 (11.3)	54 (8.4)	18 (3.1)
**Remuneration**			
No remuneration	252 (40.3)	175 (24.8)	101 (19.4)
Up to half minimum wage	165 (29.4)	129(19.2)	102 (19.3)
Between ½ to 1 minimum wage	104 (17.7)	203 (30.8)	191 (36.0)
More than the minimum wage	84 (12.6)	164 (25.2)	118 (25.3)
**Work in prison**			
No	310 (49.0)	348 (51.3)	458 (87.9)
Yes	323 (51.0)	331 (48.7)	63 (12.1)
**Age (years)**			
18–27	234 (35.6)	267 (38.1)	314 (59.9)
28–37	222 (37.3)	246 (36.2)	135 (27.1)
38–47	111 (20.2)	111 (17.1)	48 (9.2)
48–57	39 (5.0)	41 (6.8)	15 (3.0)
>57	11 (2.0)	11 (1.9)	4 (0.8)
**Cause of prison**			
Crimes against property	54 (7.2)	40 (5.6)	44 (9.8)
Drugs	161 (32.7)	61 (8.4)	56 (9.3)
Violent crimes	402 (60.1)	575 (86.0)	415 (80.9)
**Recidivist**			
No	464 (76.7)	395 (60.8)	297 (55.9)
Yes	147 (23.3)	277 (39.2)	215 (44.1)
**Time of sentence fulfilled**			
1 year	24.8 (121)	19.2 (127)	70 (356)
2 years	28 (181)	20.2 (135)	17.7 (83)
3 years	23.6 (132)	13.2 (92)	5.2 (25)
4 years or more	23.5 (178)	47.4 (310)	7.1 (34)

1 Temporary detention center.

Violent crimes (robbery, rape, murder, and bodily injury) were the main causes of imprisonment. This was more pronounced among men, because a high proportion of drug crimes (drug use and trafficking) was also found among women. Criminal recurrence was less frequent among women compared with men. We found that 76.4% of women and 52.3% of men were incarcerated for less than three years. In temporary detention centers, most of the prisoners (70.0%) were incarcerated for less than one year, and 12.3% remained at these units for more than two years.

Lifetime and 12-month prevalence of mental disorders differed between genders (Tables 2 and 3): rates of 56.1% and 21.5% were found among men, and 68.9% and 38.4% among women, respectively.

**Table 2:** Lifetime prevalence of mental disorders in prison population of the state of São Paulo, Brazil (n = 1809).

Mental Disorders	Female population		Male population					
			Prison		TDC^1^		Total	
	%	95%CI^2^	%	95%CI^2^	%	95%CI^2^	%	95%CI^2^
	%	IC 95%	%	IC 95%	%	IC 95%	%	IC 95%
**Schizophrenia**	1.9	0.9–4.0	3.2	2.1–4.7	3.9	2.4–6.4	3.4	2.5–4.7
Other psychoses^3^	2.4	1.8–3.1	1.8	0.9–3.7	2.5	1.2–5.0	2.1	1.2–3.5
**Affective disorders** 4	40.8	37.3–44.3	18.4	16.0–21.2	25.3	21.3–29.9	20.8	8.4–23.4
Mania	0.9	0.9–3.9	0.7	0.2–1.7	1.4	0.7–2.9	0.9	05–1.7
Hypomania	–	–	0.5	0.2–1.4	1.0	0.5–2.0	0.7	0.4–1.2
Depression	36.5	33.5–39.6	12.3	9.7–15.5	17. 8	14.9–21.2	14.2	12–16.8
Bipolar affective disorders	1.7	0.2–10.8	2.9	2.0–4.2	2.9	1.7–5.0	2.9	2.1–4.0
Dysthymia	6.4	4.4–9.2	4.8	2.7–8.5	5.0	3.6–6.9	4.9	3.3–7.2
**Anxious-phobic disorders** 5	50.0	45.6–54.4	32.5	29.4–35.8	40.5	35.2–46.1	35.3	32.3–38.4
Phobic disorders^6^	15.6	13.8–17.6	6.4	4.7–8.6	12.4	4.9–27.7	8.5	5.0–13.9
Panic disorder	1.1	0.9–1.3	0.5	0.1–1.8	0.8	0.2–2.5	0.6	0.2–1.4
GAD	10.4	9.3–11.7	4.9	2.4–9.8	5.4	2.2–12.4	5.0	2.9–8.6
OCD	1.2	0.8–1.8	1.1	0.4–3.3	1.5	0.5–4.8	1.2	0.6–2.8
PTSD	40.2	35.3–45.2	26.4	22.8–30.3	33.4	26.7–40.8	28.8	25.2–32.7
**Tobacco**	38.4	33.3–43.7	32.5	28.1–37.3	33.4	30.2–36.7	32.8	29.7–36.1
**Alcohol**	15.6	13.4–18.0	18.0	14.6–21.8	19.6	13.8–27.1	18.5	15.4–22.1
**Drugs**	25.2	19.2–32.2	27.1	21.6–33.3	25.3	20.7–30.4	26.5	22.5–30.8
**Severe mental disorder** 7	25.8	24.0–27.7	11.0	8.3–14.3	15.0	12.4–18.0	12.3	10.3–14.8
**Any mental disorder** (except tobacco)	68.9	63.5–73.9	54.1	49.2–59.0	60.0	55.4–64.5	56.1	52.5–59,8

1 Temporary detention center;

2 Confidence Interval;

3 Delusional Disorder, Acute Psychotic, and Schizoaffective Disorder;

4 Depression, Bipolar Disorder, Dysthymia, Hypomania, and Mania;

5 Panic, Agoraphobia, Social Phobia, Obsessive Compulsive Disorder (OCD), Generalized Anxiety Disorder (GAD), and Pos Traumatic Stress Disorder (PTSD);

6 Agoraphobia and Social Phobia;

7 Schizophrenia, Delusional Disorder, Acute Psychotic Disorder, Schizoaffective Disorder, Severe Depression, and Bipolar Affective Disorder.

**Table 3:** Twelve-month prevalence of mental disorders in prison population in the state of São Paulo, Brazil (n = 1809).

Mental Disorders	Female population		Male population					
			Prison		TDC^1^		Total	
	%	95%CI^2^	%	95%CI^2^	%	95%CI^2^	%	95%CI^2^
**Schizofrenia**	1.5	0.9–2.4	1.8	1.1–2.9	2.1	1.2–3.8	1.9	1.3–2.8
Other psychoses^3^	0.5	0.1–3.0	0.7	0.2–2.4	2.0	1.1–3.7	1.1	0.6–2.1
**Affective disorders** 4	21.0	16.8–25. 9	7.7	6.1–9.6	14.1	11.2–17.6	9.9	8.3–11.7
Mania	0.6	0.1–3.4	0.2	0.1–0.4	0.4	0.1–1.7	0.3	0.1–0.6
Hypomania	–	–	–	–	0.4	0.1–1.5	0.1	0.0–0.5
Depression	18.8	17.3–20.4	5.3	4.0–6.8	10.0	8.1–12.2	6.9	5.8–8.1
Bipolar affective disorders	1.4	0.2–8.7	1.5	0.9–2.5	2.0	0.6–6.2	1.7	0.9–3.0
Dysthymia	5.2	4.4–6.1	2.1	0.9–4.7	1.4	0.7–2.8	1.9	1.0–3.5
**Anxious-phobic disorders** 5	27.7	25.6–29.9	12.2	10.3–14.4	16.4	13.2–20.2	13.6	11.9–15.5
Phobic disorders^6^	7.3	6.6–8.0	2.4	1.2–4.6	3.3	1.5–7.4	2.7	1.6–4.6
Panic disorder	0.1	0.0–1.1	0.2	0.0–1.5	0	–	0.1	0.0–1.0
GAD	7.3	6.2–8.7	2.4	1.5–3.7	3.1	1.2–8.2	2.6	1.6–4.2
OCD	1.0	0.8–1.2	0.3	0.1–1.3	0.6	0.1–2.4	0.4	0.1–1.1
PTSD	16.1	14.9–17.4	7.8	6.3–9.8	11.1	8.3–14.6	9.0	7.5–10.7
**Tobacco**	25.4	21.1–30.1	13.4	11.3–15.7	17.4	14.4–20.9	14.8	13.0–16.7
**Alcohol**	2.4	1.4–2.7	0.5	0.1–1.6	4.8	2.3–9.6	1.9	0.9–4.1
**Drugs**	1.6	0.9–2.7	1.6	0.6–4.4	0.8	0.3–2.0	1.3	0.6–3.0
**Severe mental disorder** 7	14.7	12.0–17.9	5.1	3.8–6.8	8.7	6.9–10.9	6.3	5.1–7.7
**Any mental disorder (except tobacco)**	38.4	34.3–42.7	19.1	16.6–21.8	26.2	22.1–30.9	21.5	19.4–23.9

1 Temporary detention center;

2 Confidence Interval;

3 Delusional Disorder, Acute Psychotic, and Schizoaffective Disorder;

4 Depression, Bipolar Disorder, Dysthymia, Hypomania, and Mania;

5 Panic, Agoraphobia, Social Phobia, Obsessive Compulsive Disorder (OCD), Generalized Anxiety Disorder (GAD), and Pos Traumatic Stress Disorder (PTSD);

6 Agoraphobia and Social Phobia;

7 Schizophrenia, Delusional Disorder, Acute Psychotic Disorder, Schizoaffective Disorder, Severe Depression, and Bipolar Affective Disorder.

Considering the lifetime prevalence (Table 2), the anxious-phobic disorders were the most frequent, regardless of gender (50% women, 35.3% men) or prison regime. These were followed by affective disorders (40.8% women, 20.8% men), substance-related disorders (25.2% women, 26.5% men), and alcohol-related disorders (15.6% women, 18.5% men). Among the anxious-phobic disorders, the vast majority of prisoners had post-traumatic stress disorder (PTSD), followed by generalized anxiety and phobic disorders. Likewise, among the affective disorders, most prisoners had depressive disorder, followed by dysthymia and bipolar affective disorder.

The 12-month prevalence of mental disorders followed the same trend (Table 2). For both genders, the phobic-anxious disorders were the most prevalent (27.7% women, 13.6% men), followed by affective disorders (21%, 9.9%). Twelve-month prevalence of substance-related disorders was significantly lower than the lifetime prevalence. The most prevalent disorders among women were depression (18.8%), PTSD (16.1%), and generalized anxiety disorder (7.3%). Among men, the most prevalent were PTSD (9%), depression (6.9%) and generalized anxiety disorder (2.6%).

High prevalence rates for severe mental disorders were found among prisoners. Women were more affected (25.8% lifetime prevalence, 14.7% 12-month prevalence) than men (12.3% lifetime prevalence, 6.3% 12-month prevalence). These rates also varied according to the prison regime. Populations in temporary centers presented a higher 12-month prevalence (8.7%) than those in prison (5.1%). Similarly, 12-month prevalence rates for depression were 10.0% and 5.3% among individuals in temporary centers and in prison, respectively, and 4.8% and 0.5% for alcohol-related disorders (Table 2).

Among men, lifetime and 12-month rates of psychiatric comorbidity were 34% and 58.5%, respectively. Among women, 12-month prevalence was 46.7% and lifetime prevalence was 69.9%. For both genders, affective disorders were more associated with other disorders. Among men, the association with 12-month and lifetime phobic-anxious disorders was 85.3% and 53.7%, respectively, 87.5% and 11.1% with 12-month and lifetime psychotic disorders, 18.7% and 40.7% with alcohol-related disorders, and 2.7% and 52.3% with drug-related disorders. Among women, the association of affective disorders with 12-month and lifetime anxious-phobic disorders was 78.8% and 73.4%, respectively, 81.3% and 76.5% with 12-month and lifetime psychotic disorders, 60% and 68.3% with drug-related disorders, and 60% and 68.3% with alcohol-related disorders.

Differently from the prevalence identified in the study, the prevalence rate of self-reported mental disorders was 4.0% (95% CI: 3.3%–4.9%) among men and 3.3% (95% CI: 2.6%–4.1%) among women.

## Discussion

High prevalence rates of mental disorders among prisoners in the state of São Paulo, Brazil, were found in this study. The lifetime and 12-month prevalence for any mental disorder was, respectively, 68.9% and 38.4% among women and 56.1% and 21.5% among men. These rates are two times higher than those found in the general population of São Paulo [13] [14] [15].

This is the first large-scale epidemiological study performed with the prison population in Brazil. For diagnosis of mental disorders, we used standardized and validated instruments covering a large part of the spectrum of diseases.

Some limitations can be identified in this study, such as the exclusion of personality disorders, specific phobia, and mental retardation. Furthermore, since the interviews were performed in prisons, information such as that related to the consumption of psychoactive substances, which may result in disciplinary action, may have been omitted. Part of the sample loss in this study was due to a rebellion that occurred in the prison system of São Paulo five months before the interviews were started. This fact led to enormous tension between staff and prisoners, hindering access to some of the individuals initially screened. Nevertheless, the overall prevalence rates of mental disorders found in the current study are similar to those reported in studies involving prisoners from other countries. The exclusion of prisoners from maximum security units and hospital custody may have influenced the prevalence of mental disorders in prison. However, in a study conducted in 2008 at a Custody Hospital in Rio Janeiro [16], the authors found a similar description of subjects as ours: men, single, low-income, low education, with a high prevalence of alcohol use disorder and other drugs. The authors also found a relationship with psychotic disorders, mental retardation, and personality disorders, as expected.

A systematic literature review showed that the prevalence of mental disorders among prisoners varies from 55% to 80% [17]. The lifetime prevalence among women prisoners from Canada was 69.6% [18]. However, differently than in our study, lifetime prevalence rates higher than 80% have been reported among men [19] [20] [21]. This may be due to the fact that personality disorders were excluded from our study.

In the current study, the lifetime and 12-month prevalence rates of mental disorders were, respectively, 1.2 and 1.8 times higher among women than among men. More psychiatric morbidity among women prisoners has also been reported by other studies [22], [23]. Women are in general less likely to commit crimes, although those who commit are usually characterized by a psychopathological profile [6].

We also found that the anxious-phobic disorders were the most prevalent among prisoners, differently from other studies in which drug-related disorders were the most frequent. Nevertheless, anxious-phobic disorders have been reported as one of the most common mental disorders [21] [20] [19].

### Anxious-phobic Disorders

The high rates of anxiety-phobic disorders observed can be explained by the high prevalence rates of PTSD. Varying prevalence rates of PTSD were found among prisoners from other countries [20] [24]. In Spain, the lifetime and 12-month prevalence among men (3.5% and 0.4%) were lower than those found in this study (26.4% and 7.8%) [20]. In Australia, the 12-month prevalence rate was 25%, for both men and women. The high prevalence found in this country was attributed to the high number of stressors and traumatic events associated with imprisonment butler [24]. In São Paulo, PTSD rates may also be influenced by the instability of the prison system, which is subject to rebellions as previously mentioned.

Other studies have reported prevalence rates of generalized anxiety disorders higher than those described here [1] [25]. The exception was the study carried out with male prisoners in Iran, which identified a 12-month prevalence rate of 5.7% [21].

### Affective Disorders

Affective disorders were the second most frequent mental disorder among prisoners, with emphasis on depressive disorders. Similarly to data reported for the general population, affective disorders were three times more prevalent among women than among men. Gender-related differences in the prevalence of affective disorders have been poorly studied in the prison population, with the exception of a Canadian study, which identified a female to male ratio of 1.3 [18].

The lifetime and 12-month prevalence rates of affective disorders among men were lower than those reported by other studies [20] [21] [1], with the exception of the study conducted in Italy [19].

For both genders, the prevalence rates of depressive disorders were lower than those reported in the literature [20] [1] [25].

The prevalence of affective disorders should be analyzed carefully because the conditions of imprisonment, together with the absence of standardized diagnostic criteria, may lead to an overestimation. Incarceration may trigger symptoms similar to depression such as acute stress, grief, derealization, sleep disturbances, loss of interest and energy, low self-esteem, worrying, and anxiety [9].

### Drug-related Disorders

For both genders, the lifetime prevalence of drug-related disorders was higher than those reported by studies performed in the general population [14] [13]. Unlike those, which have shown higher rates of drug-related disorders among men, we found no gender-related differences.

In general, the rates of drug- or alcohol-related disorders were lower than those found in other studies of prison populations [26] [20].

The differences in prevalence found in the studies may be explained by the particularities of each country, such as the criminalization and the cultural pattern of drug use [23], and the access to these substances in prisons [9]. The low prevalence rate found in our study may be associated with the controlling role played by organized groups of inmates. These groups were created in the prisons of São Paulo to regulate coexistence among inmates with the purpose of self-protection [27]. The consumption of hard drugs, because of its destabilizing character, may have required stricter disciplinary codes imposed by these groups, resulting in reduced drug use.

### Severe Mental Disorders

The high prevalence rates of severe mental disorders among prisoners contrast with estimated general population rates. While rates vary between 0.4% and 7.7% in the general population [28], we found 12-month prevalence rates of 14.7% and 6.9% among women and men prisoners, respectively. These results are similar to those of other studies of prison populations. Some type of severe mental disorder was found in 15% of women and 5% of men in prisons in New York City [29], and in 17.7% of women and 7.8% of men in prisons in Florida, USA [30].

The clinical conditions associated with severe mental illness are chronic and may constitute a risk of suicide and significant psychosocial damage, requiring specialized services [18]. Moreover, these inmates are frequent victims of discrimination and humiliation and may pose a risk for the stability in the prison given the behavioral changes associated with psychiatric disorders [31].

### Psychiatric Comorbidity

We found high rates of psychiatric comorbidity, particularly among women prisoners. These results agree with those of other studies, which have shown rates from 50% to 90% [6]. The high rate of comorbidity, which has been linked to the violent and offending profile of inmates [21] [26] [20], reinforces the impact and severity of mental disorders in prisons.

### Penitentiary System

Similarly to other studies, we found that severe mental disorders, alcohol-related disorders, and depression were more frequent among prisoners in temporary regime (TDC) than among those in closed regime (prisons) [6]. The prisoner under a temporary regime is usually waiting to be transferred to a prison. Thus, this difference in prevalence may be due to the transfer of prisoners to psychiatric treatment centers instead of to prisons [9]. On the other hand, the fact of being under a temporary regime may be very distressing due to several destabilizing experiences, such as the new condition of being incarcerated, the fact of having to deal with judicial processes and uncertainties about the future [31].

### Issues of Incarceration

The main causes that explain the high prevalence of mental disorders in prison populations are the stressful conditions imposed in the execution of the sentence and prior morbid conditions that make people more likely to commit crimes [32].

Regardless of whether mental disorders are the cause or consequence of the prison, the conditions of imprisonment with its inherent stressors do not contribute to preserving mental health and do not favor the treatment of individuals with more severe or chronic mental disorders [24]. Furthermore, the prison systems do not always have adequate resources for health care. Complicating factors for the evaluation and treatment of mental disorders in prison have been identified, such as the limited validity of the records and observations on prisoner admission, inadequate transmission of information between prison professionals, identification of certain symptoms (e.g., anxiety and aggression) over others (e.g., depressive or psychotic symptoms) and the absence of tools to support diagnosis [18].

The prisoners may not recognize their own illness, and thus fail to seek psychiatric treatment. This fact impairs initiatives towards promoting mental health and treatment in prison. In this study, we observed very low rates of self-reported mental disorders (4.0% among women, 3.3% among men), which may be explained by insight impairment, by the stigma associated with mental illness and by the fear of being transferred to custodial hospitals [19].

Based on this information, measures to improve health conditions of prisoners have been proposed. They include improved screening methods for mental disorders, prison staff training, reducing the ratio between officers and mental health professionals and prisoners, confining one prisoner per cell, increasing encounters between prisoners and their families, offering educational programs, work and exercise in prisons, and applying policies against sexual assault, among others [2].

## Conclusions and Recommendations

This is the first large-scale epidemiological study performed with the prison population in Brazil, revealed high rates of psychiatric disorders among men and women. Many similarities, as well as some differences, were found between our results and those of studies conducted in other countries. The differences observed are more likely due to the peculiarities of the prison systems in each country than to the diagnostic criteria adopted in the studies. This fact reinforces the importance of conducting such studies as part of planning and development of appropriate policies for the particular mental health needs of specific prison populations.
